# Two new species of the genus *Panorpa* Linnaeus (Mecoptera, Panorpidae) from Yunnan, China

**DOI:** 10.3897/zookeys.587.7674

**Published:** 2016-05-10

**Authors:** Ji-Shen Wang, Bao-Zhen Hua

**Affiliations:** 1 Key Laboratory of Plant Protection Resources and Pest Management, Ministry of Education, Entomological Museum, Northwest A&F University, Yangling, Shaanxi 712100, China

**Keywords:** Biodiversity, Hengduan Mountains, Mount Daxueshan, scorpionfly, taxonomy

## Abstract

Two new scorpionfly species, *Panorpa reflexa* **sp. n.** and *Panorpa parallela* **sp. n.**, are described and illustrated from Yunnan Province, China. *Panorpa reflexa* can be readily differentiated from its congeners by the 3-shaped parameres in male genitalia. *Panorpa parallela* is unique for its parallel parameres in male genitalia. The number of *Panorpa* species is raised to four in Yunnan Province, and to 113 throughout China.

## Introduction

Panorpidae are the largest family in Mecoptera, and currently consist of approximately 400 species in six genera worldwide (Hu et al. 2015). Panorpids are commonly called scorpionflies, because the genitalia of their males are globular and recurved over the back, resembling the tail of a scorpion (Byers and Thornhill 1983; Bicha et al. 2015). Scorpionflies mostly live in humid, high-elevated and vegetation-rich habitats (Byers and Thornhill 1983; Byers 2002a). Males of many scorpionflies offer nuptial gifts to the female during courtship and copulation, and use the clamp-like notal organ on the third tergum to seize the anterior edge of the wings of the female (Zhong and Hua 2013a). These insects are frequently considered to represent ideal models for the study of mating systems and behavior in insects (Ma and Hua 2011a; Zhong et al. 2015a). However, the males of *Furcatopanorpa longihypovalva* (Hua & Cai, 2009) lack a notal organ, and assume an unusual O-shaped position during copulation (Zhong et al. 2015b).

*Panorpa* Linnaeus, 1758 is the most speciose genus in Panorpidae, consisting of approximately 252 species worldwide and 111 species in China to date (Esben-Petersen 1921; Carpenter 1945; Byers 1970; 2002b; Chou et al. 1981; Fu and Hua 2009; Zhang and Hua 2012; Zhong and Hua 2013b). *Panorpa* differs from *Neopanorpa* Weele, 1909 and *Leptopanorpa* MacLachlan, 1875 by the vein 1A ending at or beyond the level of the origin of Rs, in addition to genital features (Cheng 1957). Based on morphological and molecular data (Misof et al. 2000; Ma et al. 2011, 2012; Hu et al. 2015), the genus *Panorpa* has been confirmed to be a paraphyletic group. Recently, three genera have been established from *Panorpa* Linnaeus: *Sinopanorpa* Cai & Hua in Cai et al. 2008 (3 spp.), *Furcatopanorpa* Ma & Hua, 2011 (1 sp.) and *Dicerapanorpa* Zhong & Hua, 2013 (8 spp.).

Yunnan is a province in southwestern China and well-known for its richness in biodiversity (Yang et al. 2004). Historically, five species of *Panorpa* were recorded from Yunnan. Recently, *Panorpa kimminsi* Carpenter, 1948, *Panorpa triclada* Qian & Zhou, 2001, and *Panorpa tjederi* Carpenter, 1938 were transferred to *Dicerapanorpa* Zhong & Hua, 2013. Consequently, only two species, *Panorpa issikiana* Byers, 1970 and *Panorpa kunmingensis* Fu & Hua, 2009, remain in the genus *Panorpa* from Yunnan. In our recent survey in the Mount Daxueshan, the southernmost prolongation of the Hengduan Mountains, numerous scorpionfly specimens were collected and determined to belong to two undescribed species of *Panorpa* Linnaeus, raising the number of *Panorpa* species to four in Yunnan Province, and to 113 throughout China.

## Material and methods

Specimens examined in this study were captured with collecting nets and temporarily preserved in 75% ethanol. After observation and initial measurements, some type materials were pinned as permanent preservation and deposited in the Entomological Museum, Northwest A&F University, China (NWAU).

Measurements of the right wings of 20 males and 20 females of the two new species were made with a vernier calliper, calculated with Microsoft Excel 2010 and are presented as mean ± SD (standard deviation). Some genitalia were macerated in 10% NaOH solution for 3 minutes and then rinsed with tap water. Photographs were taken with a Nikon D7000 digital camera, and further treated with Adobe Photoshop CS4. Dissections and observations were made under a Nikon SMZ 1500 microscope. The abdominal segments are described as abbreviates, e.g., A1 is the first segment.

## Description

### Panorpa reflexa sp. n.

Taxon classificationAnimaliaMecopteraPanorpidae

http://zoobank.org/3264DA6D-00F6-4302-9AE0-302C058641B1

Figs 1 , 2 , 3 , 4A

#### Type material.

**Holotype: CHINA: Yunnan Province**: ♂, Mt. Daxueshan [大雪山] (24°11.27'N, 99°37.35'E), 2000 m, Yongde County [永德县], 21 Aug. 2015, leg. Ji-Shen Wang. **Paratypes.** 31♂♂48♀♀, same data as the holotype, 21–23 Aug. 2015.

#### Diagnosis.

The new species can be recognized by the following features: 1) dorsum of body with one broad pale longitudinal stripe mesally; 2) head yellowish brown with the ocellar triangle and the postvertex black; 3) wings hyaline with a greatly reduced pterostigmal band; 4) hypandrium greatly shortened, with hypovalves only reaching the basal third of the gonocoxites; and 5) parameres 3-shaped.

#### Description of male.

**Figure 1.**
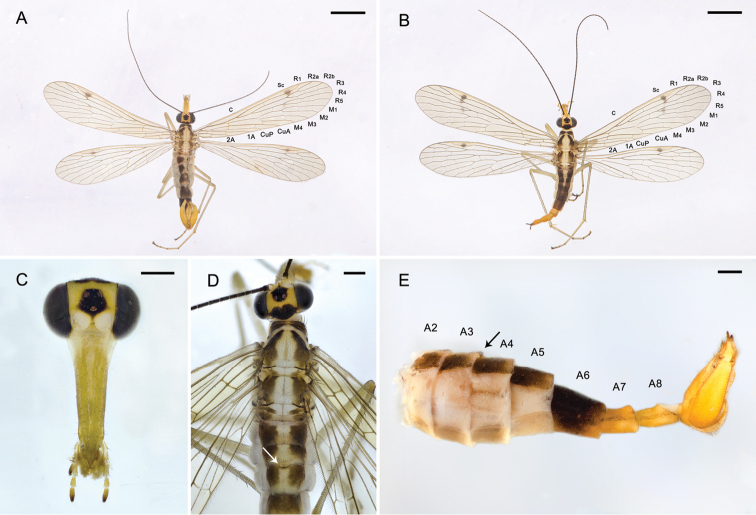
Adults of *Panorpa reflexa* sp. n. **A** Habitus of male, dorsal view **B** habitus of female, dorsal view **C** head of male with antennae removed, frontal view **D** dorsum of male **E** abdomen of male, lateral view. Arrows show the notal organ on tergum III. Scale bars: 2.5 mm (**A, B**); 0.5 mm (**C**–**E**).

*Head*. Head mostly yellow. A black pattern on postvertex, transverse, shallowly notched on anterior margin, and laterally adjacent to compound eyes; another black pattern around ocellar triangle, almost pentagonal, with its anterior margin extending to the upper border of the light yellow antennal sockets. Antennal scape brown, pedicel dark brown, flagellum black with 38–42 segments. Rostrum unevenly yellowish brown with genae pale. Maxillary and labial palps yellowish with distal segments darkening toward the apex (Fig. 1C).

*Thorax*. Pronotum brown, with 6–10 stout setae along its anterior margin. Meso- and metanotum brown with a broad pale longitudinal stripe and a narrow brown mesal line (Fig. 1D). Pleura pale yellow. Legs light brown with coxae pale yellow and distal tarsomeres brown.

*Wings*. Forewing length 10.40 ± 0.37 mm, width 2.40 ± 0.17 mm. Pterostigma light brown with dense microtrichia. Wing membrane hyaline, devoid of markings except the greatly reduced brown pterostigmal band, which forms an irregular spot at vein R_1_ and extending to vein M_1_ as a series of discontinuous spots. Vein 1A ending at the hind margin beyond the level of the origin of Rs. Hindwing length 9.47 ± 0.36 mm, width 2.28 ± 0.11 mm, similar to forewings, but with the pterostigmal band more degenerated (Fig. 1A).

*Abdomen*. Terga I–V brown, with a pale longitudinal mesal stripe connected with the thoracic stripe anteriorly and narrowing posteriorly. Notal organ on the posterior margin of tergum III slightly developed and covering the acute dorsal process of tergum IV (Fig. 1D). Sterna I–V light brown, pleura pale. A6 blackish brown with distal third yellowing gradually, cylindrical, without anal horns. A7 and A8 yellowish orange, with faint brown textures laterally; A7 nearly cylindrical, A8 slightly constricted basally and beveled apically (Fig. 1E).

**Figure 2.**
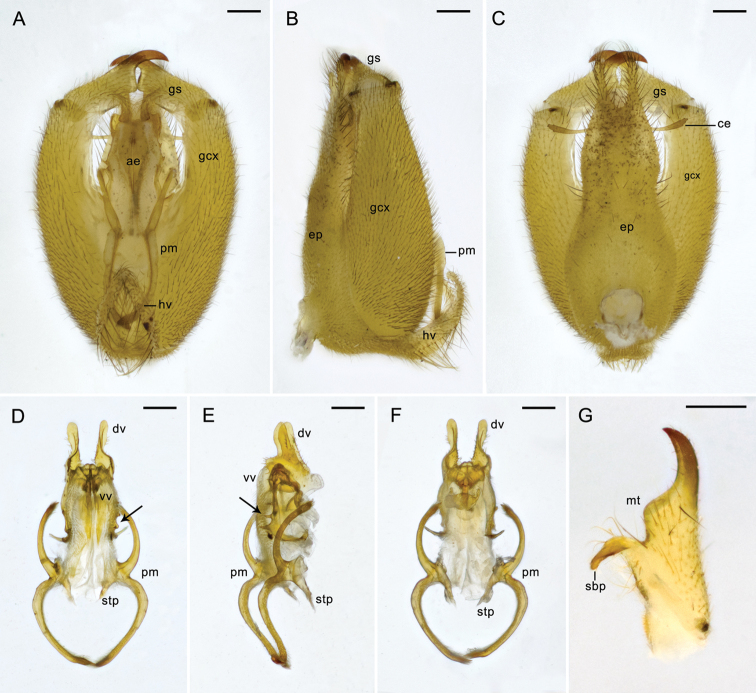
Male genitalia of *Panorpa reflexa* sp. n. **A**–**C** Genital bulb in ventral, lateral, and dorsal views **D–F** aedeagal complex in ventral, lateral, and dorsal views, arrows pointing to the teeth along the joint edges of dorsal and ventral aedeagal valves **G** gonostylus, dorsal view. **ae** aedeagus; **ce** cercus; **dv** dorsal valve; **ep** epandrium; **gcx** gonocoxite; **gs** gonostylus; **hv** hypovalve; **mt** median tooth; **pm** paramere; **sbp** subbasal process; **stp** stalk of paramere; **vv** ventral valve. Scale bars: 0.2 mm.

*Male genitalia*. Genital bulb yellowish orange, oval, slightly flat in lateral view (Fig. 2). Epandrium (tergum IX) extending beyond the apex of gonocoxite, slightly constricted midway, distal half tapering toward the apex, with a deep U-shaped terminal emargination and forming two parallel digital processes (Fig. 2C). Cerci clavate. Hypandrium (sternum IX) greatly shortened, extending only to the basal third of gonocoxites, hypovalves bearing long bristles along inner margins (Fig. 2A). Gonostylus shorter than half the length of gonocoxite, outer margin slightly concaved, inner margin with a blunt median tooth and a curved subbasal process (Fig. 2G). Parameres 3-shaped; both arms nearly half the length of aedeagus, with acute tips, and bearing numerous microtrichia along inner margins; anterior arms stretched under hypovalves. (Fig. 2A, B). Ventral valves of aedeagus thick, slightly sclerotized with apexes rounded, bearing numerous soft setae; dorsal valves strongly sclerotized, with many marginal spines and prominent, flattened apexes, exceeding to the base of gonostylus; the joint edge of dorsal and ventral valves rolled ventrad, with three acute teeth ventrally, and one long spine dorsolaterally (Fig. 2D–F).

#### Description of female.

Similar to males in general appearance. The ocellar pattern more rounded than that of males; wings with pterostigmal band more developed (Fig. 1B). Forewing length 11.10 ± 0.35 mm, width 2.48 ± 0.13 mm; hindwing length 10.17 ± 0.27 mm, width 2.31 ± 0.11 mm, similar to forewings.

**Figure 3.**
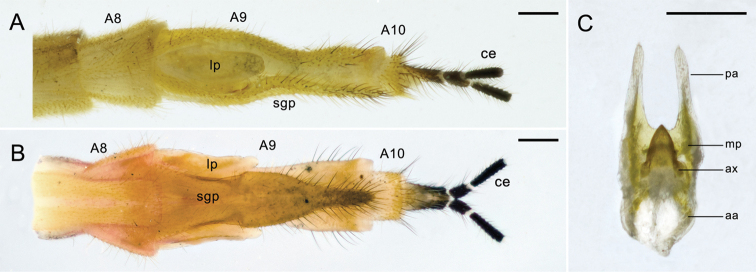
Female terminalia of *Panorpa reflexa* sp. n. **A** and **B** Terminalia in lateral and ventral views **C** genital plate, ventral view. **aa** anterior arm; **ax** axis; **ce** cercus; **lp** lateral plate; **mp** main plate; **pa** posterior arm; **sgp** subgenital plate. Scale bars: 0.2 mm.

*Female genitalia*. A9 nearly twice the length of A8. Subgenital plate accompanied with two lateral plates, which slightly beyond half the length of the main part; main part with basal half trapezoid, distal half vase-shaped and bearing long thick setae marginally (Fig. 3A, B). Genital plate small, shorter than half of subgenital plate; axis short, entirely concealed in the main plate, posterior apex acute; anterior arms shorter than main plate, curved entad; posterior arms longer than main plate, straight, narrowed toward the apex, slightly convergent (Fig. 3C).

#### Etymology.

The specific epithet is derived from the Latin *reflex*- (turned back, reflected), referring to the anterior arms of the paramere in male genitalia being reflected, and not directed caudad as usual.

#### Distribution.

China (Yunnan Province).

#### Comparisons.

*Panorpa reflexa* sp. n. is similar to *Panorpa decolorata* Chou & Wang, 1981 from Shaanxi Province, *Panorpa filina* Chou & Wang, 1987 from Hunan Province, *Panorpa waongkehzengi* Navás, 1935 from Jiangxi Province, China in abdominal morphology, but differs from the latter three species by the males of *Panorpa reflexa* with the paramere 3-shaped and its anterior arm directed cephalad (cf. parameres are directed caudad, whether branched or not).

*Panorpa reflexa* resembles *Panorpa guttata* Navás, 1908 from Sichuan Province, China in the greatly reduced pterostigmal band in wings, but can be easily distinguished from the latter by the black ocellar triangle and postvertex (cf. vertex is uniformly colored).

### Panorpa parallela sp. n.

Taxon classificationAnimaliaMecopteraPanorpidae

http://zoobank.org/243661E8-7438-4B26-B3C6-5D904B7CDBCD

Figs 4B , 5 , 6 , 7

#### Type material.

**Holotype: CHINA: Yunnan Province**: ♂, Mt. Daxueshan [大雪山] (24°11.27'N, 99°37.35'E), 2000 m, Yongde County [永德县], 21 Aug. 2015, leg. Ji-Shen Wang. **Paratypes.** 22♂♂32♀♀, same data as the holotype, 21–23 Aug. 2015.

#### Diagnosis.

This new species resembles *Panorpa reflexa* sp. n. in appearance, but can be readily differentiated from the latter by the following characters: 1) head yellowish brown, with ocellar triangle black (cf. head yellow with two black patterns, one on dorsum and the other one around ocellar triangle); 2) forewing with a faint apical band (cf. apical band absent); 3) hypandrium with elongated basal stalk (cf. basal stalk extremely shortened); 4) parameres in male genitalia simple and protruding caudad, almost parallel (cf. 3-shaped).

#### Description of male.

*Head*. Head muddy yellow with ocellar triangle black. Antennal socket light yellow, scape yellowish brown, pedicel dark brown, flagellum black and with 39–42 segments. Rostrum unevenly muddy yellow with genae pale, subgenae brown. Maxillary and labial palps yellowish brown with distal segments darkening toward the apex (Fig. 5C).

**Figure 4.**
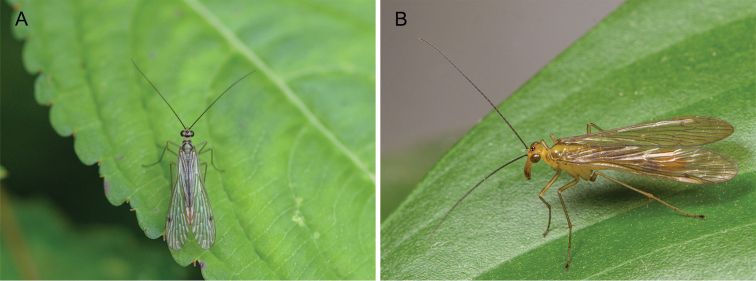
Male adults in the field. **A** *Panorpa reflexa* sp. n. **B** *Panorpa parallela* sp. n.

**Figure 5.**
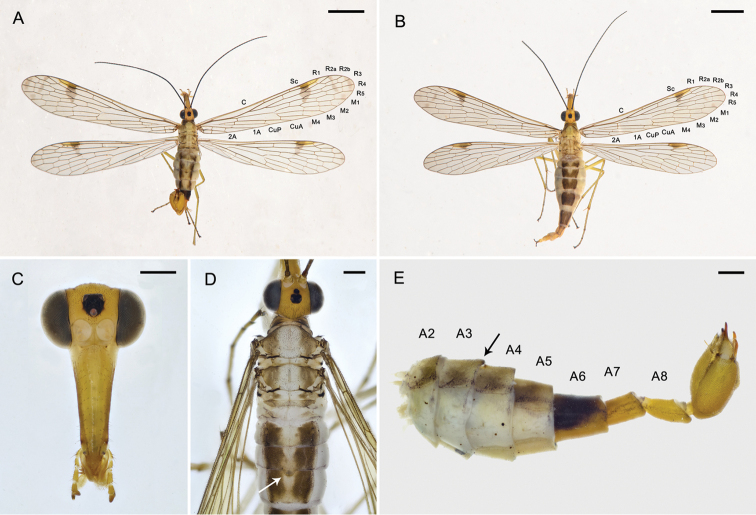
Adults of *Panorpa parallela* sp. n. **A** Habitus of male, dorsal view **B** habitus of female, dorsal view; **C** head of male with antennae removed, frontal view; **D** dorsum of male; **E** abdomen of male, lateral view. Arrows show the notal organ on tergum III. Scale bars: (**A, B**): 2.5 mm; (**C–E**): 0.5 mm.

*Thorax*. Pronotum brown, bearing 4–6 stout setae along its anterior margin. Meso- and metanotum light brown with a broad yellowish longitudinal stripe and a faint brown mesal line (Fig. 5D). Pleura pale brown. Legs yellow, with distal tarsi deep brown.

*Wings*. Forewing length 10.81 ± 0.51 mm, width 2.37 ± 0.10 mm, membrane hyaline, pterostigma reddish brown with dense microtrichia. Pterostigmal band brown, reduced to an irregular spot with acute hind apex, extending to vein R_2+3_, even to M_1_ in some individuals. Apical band faint, degenerated as two irregular stripes, very faint in a few individuals. Vein 1A ending almost at the level of the origin of Rs. Hindwing length 9.98 ± 0.40 mm, width 2.19 ± 0.08 mm, similar to forewings but pterostigmal band and apical band more degenerated (Fig. 5A).

*Abdomen*. Terga I–V sordidly brown, with a yellowish longitudinal mesal stripe, which is weakened and forms several continuous or discontinuous triangular spots at each tergum; pleura pale, sterna light brown. Notal organ on the posterior margin of tergum III slightly developed, covering the acute dorsal process of tergum IV (Fig. 5D). A6 dark brown dorsally and yellowish ventrally, without anal horns. A7 and A8 yellowish orange, with some faint brown textures along lateral surfaces; A7 cylindrical, A8 constricted basally and beveled apically (Fig. 5E).

**Figure 6.**
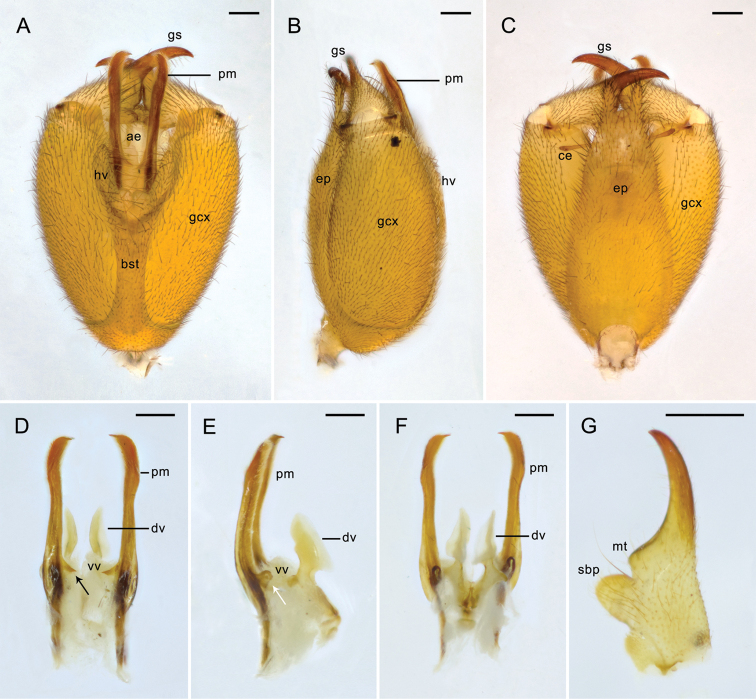
Male genitalia of *Panorpa parallela* sp. n. **A–C** Genital bulb in ventral, lateral, and dorsal views **D–F** aedeagal complex in ventral, lateral, and dorsal views, black and white arrows show the inner teeth and dorsal process of paramere, respectively **G** gonostylus, ventral view. **ae** aedeagus; **bst** basal stalk of hypandrium; **ce** cercus; **dv** dorsal valve; **ep** epandrium; **gcx** gonocoxite; **gs** gonostylus; **hv** hypovalve; **mt** median tooth; **pm**, paramere; **sbp** subbasal process; **vv** ventral valve. Scale bars: 0.2 mm.

*Male genitalia*. Genital bulb yellowish orange, oval (Fig. 6A–C). Epandrium (tergum IX) extending beyond the apex of gonocoxite, broad basally and tapering toward the apex, with a deep U-shaped terminal emargination (Fig. 6C). Cerci brown. Hypandrium (sternum IX) Y-shaped, with a narrow elongated basal stalk and splitting into paired hypovalves distally, extending to four-fifths of gonocoxite (Fig. 6A). Posteroventral margin of gonocoxite with a flat triangular process. Gonostylus slightly concaved along outer margin, inner margin with a blunt median tooth and an oval subbasal process, a long bristle rising between them (Fig. 6G). Parameres reddish brown, stick-like, extending far beyond the median tooth of gonostylus, approximately parallel, apexes acute and curved convergently, distal third with numerous microtrichia; inner margin with a pointed tooth subbasally, dorsal margin with a flat process next to the subbasal tooth (Fig. 6D–F). Ventral valves of aedeagus poorly developed, membranous; dorsal valves sclerotized, separated, with thin neck-like stalks, distal parts swollen and flatiron-shaped (Fig. 6D–F), extending nearly to the base of gonostylus.

#### Description of female.

Similar to males in coloration and patterns (Fig. 5B). Forewing length 11.72 ± 0.41 mm, width 2.58 ± 0.15 mm; hindwing length 10.76 ± 0.52 mm, width 2.33 ± 0.15 mm, similar to forewings.

**Figure 7.**
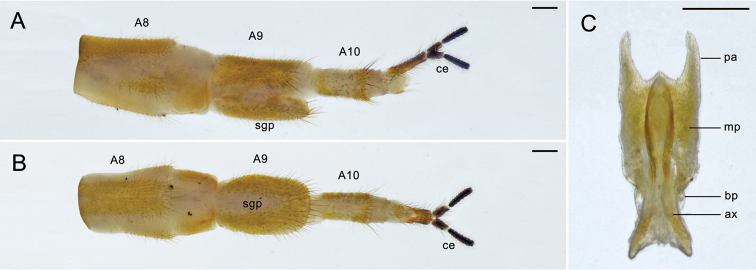
Female terminalia of *Panorpa parallela* sp. n. **A, B** Terminalia in lateral and ventral views; **C** genital plate, ventral view. **ax** axis; **bp** basal plate; **ce** cercus; **mp** main plate; **pa** posterior arm; **sgp** subgenital plate. Scale bars: 0.2 mm.

*Female genitalia*. A9 slightly shorter than A8. Subgenital plate long elliptic (Fig. 7A, B). Genital plate with main plate oblong and intensely constricted at base, posterior margin with a triangular mesal prominence; basal plate slightly wider than axis but narrower than main plate; axis elongated beyond the main plate, anterior third divergent widely, posterior third long elliptic with an acute apex; posterior arms almost half the length as main plate, broad basally and narrowed toward the apex, almost parallel (Fig. 7C).

#### Etymology.

The specific epithet is derived from the Latin *parallel*- (parallel), referring to the parallel parameres in male genitalia.

#### Distribution.

China (Yunnan Province).

#### Comparisons.

*Panorpa parallela* sp. n. is similar to *Panorpa rufostigma* Westwood, 1842 from Europe in the reddish pterostigma, but can be recognized by its greatly reduced wing markings (cf. wing markings well-developed).

*Panorpa parallela* resembles *Panorpa chengi* Chou, 1981 from Shaanxi Province, China in body coloration, especially the pale-brown thoracic terga, but can be differentiated from the latter by the reddish pterostigma and the wing pattern (cf. pterostigma indistinct, wings devoid of markings).

The two new species, *Panorpa reflexa* and *Panorpa parallela*, resemble each other in general appearance at first glance. In living animals, the wings are held in close contact along the mid-line over the abdomen, and in the same plane at repose (Fig. 4). This condition is different for most species of *Panorpa*, the wings of which are mostly divergent and kept in a V-shape over the abdomen at repose.

#### Habitat.

**Figure 8.**
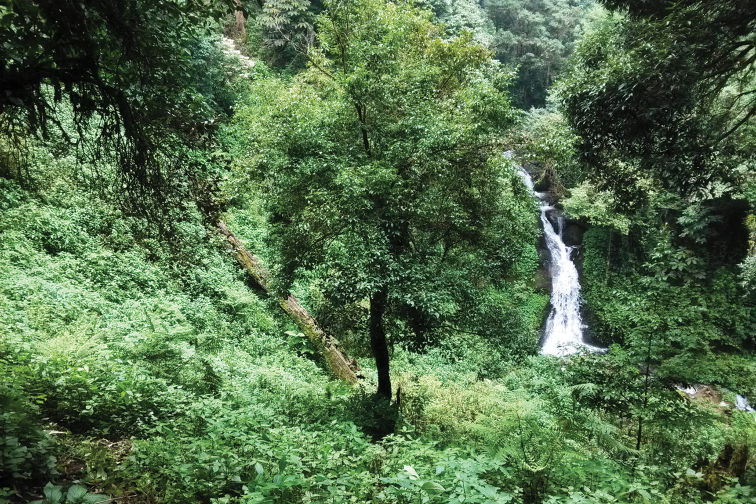
Habitat of the type locality.

In the type locality, Mount Daxueshan, these two species share the same habitat among several mountain valleys with streams around an elevation of 2000 m. Suitable microhabitats lie mostly in a slope surrounded by evergreen broad-leaved forests and with dense herbaceous groundcover (Fig. 8). In the daytime of August, these valleys are mostly overspread with mist, receiving little direct sunlight, and the temperature ranges approximately from 16 to 22 °C.

## Supplementary Material

XML Treatment for Panorpa reflexa

XML Treatment for Panorpa parallela
